# Case Report: Considerations of nocturnal ventilator support in ROHHAD syndrome in chronic care of childhood central hypoventilation with hypothalamus dysfunction

**DOI:** 10.3389/fped.2022.919921

**Published:** 2022-08-31

**Authors:** Rui Zhao, Xiaosong Dong, Zhancheng Gao, Fang Han

**Affiliations:** Department of Pulmonary and Critical Care Medicine, Peking University People’s Hospital, Beijing, China

**Keywords:** ROHHAD syndrome, hypoventilation, obesity, hypothalamic dysfunction, noninvasive positive pressure ventilation

## Abstract

Rapid-onset obesity with hypothalamic dysfunction, hypoventilation, and autonomic dysregulation (ROHHAD) is a rare life-threatening disorder that can occur during childhood. All children with ROHHAD develop alveolar hypoventilation during wakefulness and sleep. The key treatment for these patients is the optimization of oxygenation and ventilation. Here, we report the case of a 5-year-old girl with suspected ROHHAD, with rapid weight gain, breathing cessation, decreased height, hypoventilation, central hypothyroidism, hyperprolactinemia, and absolute deficiency of growth hormone, and negative PHOX2B sequencing results. The presentation met the diagnostic criteria for ROHHAD syndrome. During the 5-year follow-up, she presented with progressive deterioration of the function of the hypothalamus and respiratory center, hypoxemia (PO_2_ < 60 mmHg), and hypercapnia [transcutaneous carbon dioxide (TcPCO_2_) > 70 mmHg] during the first two cycles of N3 sleep with a poor response to ventilatory support. Early diagnosis and application of non-invasive positive pressure ventilation during sleep can improve the quality of life and outcomes of patients with ROHHAD, and polysomnography and TcPCO_2_ should be repeated every 3–6 months to follow the progress and regulate ventilator support. Multidisciplinary care is crucial for the successful management of these patients.

## Introduction

Rapid-onset obesity with hypothalamic dysfunction, hypoventilation, and autonomic dysregulation (ROHHAD) syndrome is a rare disorder commonly diagnosed in childhood that features disrupted respiratory control, autonomic nervous system regulation, and hypothalamic function (1). Young patients exhibit breathing abnormalities, snoring, pauses in breathing, and shallow breathing during sleep. Formerly called late onset-central hypoventilation syndrome (LO-CHS), it was recognized as a special type of congenital central hypoventilation syndrome until 2007 when Ize-Ludlow named it “ROHHAD” because of the existence of other features such as dramatic and rapid weight gain, central hypoventilation, hypothalamic dysfunction, abnormality of the endocrine system and negative PHOX2B sequencing results (2–4). No specific cause has been identified for ROHHAD, and etiological studies are still underway (2, 5, 6).

In this case report, we describe a 5-year-old Chinese girl with ROHHAD syndrome who underwent treatment over time to emphasize the importance of early diagnosis and nocturnal ventilatory support in such patients.

## Case report

The girl was admitted at the age of 5 years and 6 months with progressive abnormal weight gain for 3 years along with snoring, sleep apnea, and cyanosis for 6 months. The patient was born at full-term *via* cesarean section, with a 3.55 kg birth weight and 51 cm length, which are normal parameters according to Chinese neonatal birth conditions. The neonatal and infancy periods were unremarkable. Since 2.5 years of age, she has demonstrated rapid weight gain due to overeating, and at age 5, she underwent a sleep study showing gasping, snoring, breathing cessation during sleep, and nocturnal enuresis. At that time, the patient was diagnosed with hypothyroidism and treated with levothyroxine sodium (12.5 mg). She developed behavioral changes, including aggressive behavior, episodes of anxiety, and nervousness. She showed no improvement with thyroid hormone replacement therapy.

**FIGURE 1:**
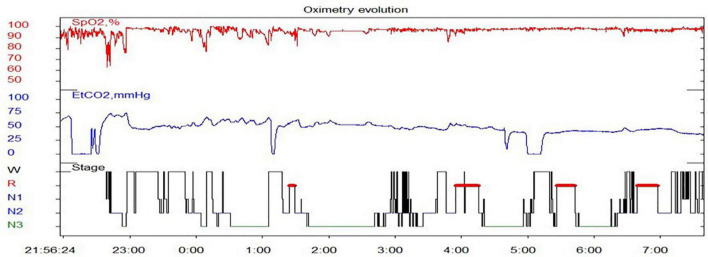
Nocturnal pulse oximetry, TcPCO_2_ evolution, and sleep stage presentation in PSG, starting at 1 a.m. of the BPAP pressure titration. Hypoventilation was seen (SpO_2_ between 60 and 98%; PCO_2_ maximum 75 mmHg) for 4 h during the night, accompanied by sleep structure disruption (recurrent arousal and inability to deep sleep). Sleep stage: W, wake; R, REM stage, N1, NREM stage 1; N2, NREM stage 2; N3, NREM stage 3.

On physical examination, she was obese with a weight of 30 kg and height of 122.5 cm; the results of other physical examinations were unremarkable. Laboratory tests showed severe hypoxia (SaO_2_ 91%, oxygen 4 L/min) and hypercapnia (PCO_2_ 50 mmHg) when awake. During sleep, the hypoxia became even more pronounced (minimum SaO_2_ 60%, 4–5 L/min oxygen) without arousal reactions. Table 1 shows low free triiodothyronine (T3), low thyroxine (T4), and low thyroid-stimulating hormone (TSH) levels with a daily supplement of 12.5 mg levothyroxine, indicating central hypothyroidism. Hyperprolactinemia was also observed. Laryngoscopy, chest radiography, echocardiography, and brain magnetic resonance imaging of the hypothalamus and pituitary gland were unremarkable. Pulmonary hypertension was not observed. As alveolar hypoventilation was highly suspected, nocturnal polysomnography (PSG) with transcutaneous carbon dioxide pressure (TcPCO_2_) was performed. No obstructive or central apnea-hypopnea events were found, and the apnea-hypopnea index was 0 events/h. Due to severe hypoxemia (SpO_2_ 60%) and hypercapnia (PCO_2_ 75 mm Hg), the PSG had to be interrupted and restarted with bi-level positive airway pressure (BPAP) titration *via* a nasal mask (Figure 1). Normal tidal volume was guaranteed in spontaneous/timed mode with inspiratory positive airway pressure (IPAP) of 17 cmH_2_O, expiratory positive airway pressure (EPAP) of 5 cmH_2_O, and a frequency of 18 times per minute.

**TABLE 1:** Endocrinologic abnormalities of pituitary-thalamus function.

Hormone		Age of presentation	Lab findings	Normal range
Thyroid function test	T3 (pmmol/L)	5 years and 6 months	3.08	3.4–6.5
	T4 (pmmol/L)	5 years and 6 months	8.64	10.2–21.8
	TSH (pmmol/L)	5 years and 6 months	0.28	0.3–3.6
Prolactin (ng/ml)		6 years and 2 months	64.93	5.18–26.53
IGF-1 (ng/ml)		9 years and 11 months	74.2	180–800
GH provocation test	0 h (μg/L)	9 years and 11 months	0.12	Peak GH > 10 μg/L
	0.5 h (μg/L)	9 years and 11 months	0.19	Peak GH > 10 μg/L
	1 h (μg/L)	9 years and 11 months	0.14	Peak GH > 10 μg/L
	1.5 h (μg/L)	9 years and 11 months	0.14	Peak GH > 10 μg/L

T3, free triiodothyronine; T4, thyroxine; TSH, thyroid-stimulating hormone; IGF-1, insulin-like growth factor-1; GH, growth hormone.

Molecular analysis for PHOX2B was inconsistent with congenital central hypoventilation syndrome.

The presence of rapid and early onset obesity, alveolar hypoventilation, hyperprolactinemia, central hypothyroidism, behavioral disorders, and absence of PHOX2B gene mutations is consistent with ROHHAD syndrome. Nocturnal BPAP ventilation and daily levothyroxine supplementation were prescribed to the patient during long-term follow-up.

**FIGURE 2:**
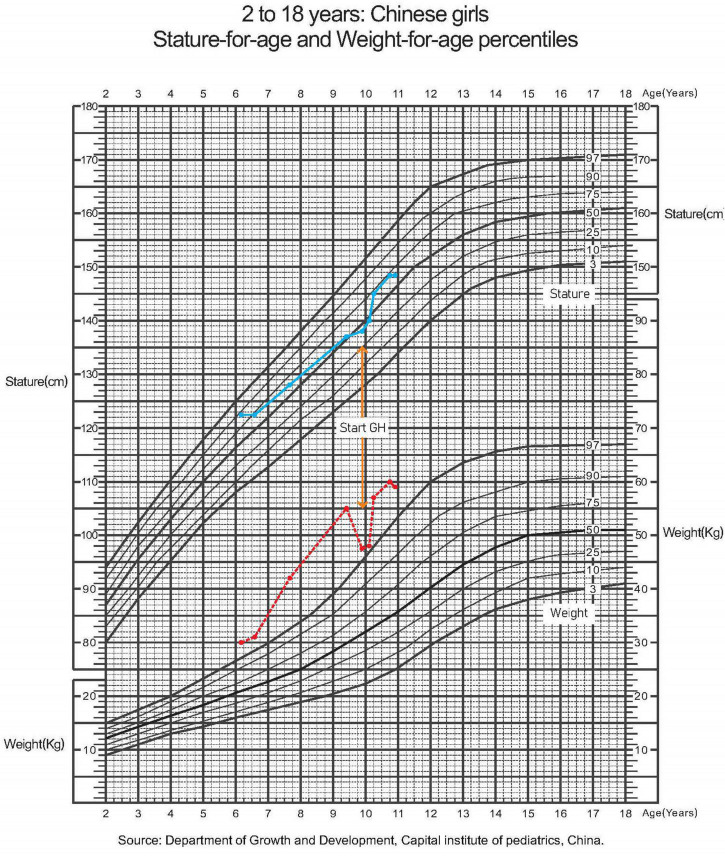
A growth curve showing stature and weight for age. Dotted line in red, weight; solid line in blue, height.

At the age of 7 years, the girl was intubated in the emergency room because of severe pneumonia and was under mechanical ventilation for 45 days. When she was 8 years old, her height growth slowed, while her weight gain did not reach 10 kg per year. The growth curves of the patient are shown in Figure 2. As illustrated in Table 1, further endocrinological investigations confirmed an absolute deficiency of growth hormone (GH) and low-level insulin-like growth factor-1. A negative result of the GH provocation test indicated dysfunction of the hypothalamic-pituitary axis. No evidence of adrenal insufficiency or diabetes insipidus was found.

Her height increased by 2 cm and her weight increased by 0.5 kg in 2 months after she received regular subcutaneous injections of GH and vitamin D prescribed by an endocrinologist.

Her liver and kidney function, electrolytes, complete blood count, and urine test results were normal. Ultrasonography findings of the breast and pelvis were normal. Chest and abdominal computed tomography scans showed no evidence of neural crest tumors.

**FIGURE 3:**
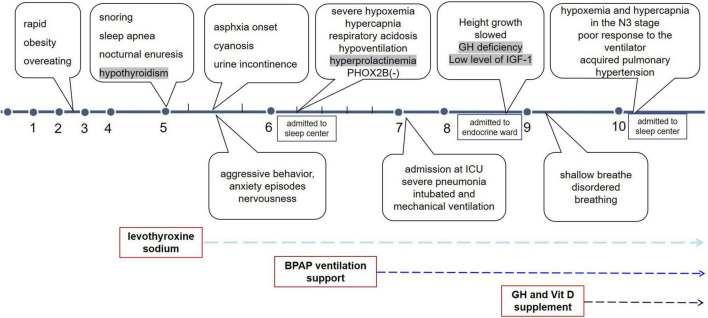
Timeline of symptoms and progression.

When she was 10 years and 2 months old, she visited our sleep center again with the complaint of “forgetting to breathe” and visible disordered breathing (weak and strong alternating episodes) even when she was awake. Cyanosis and urinary incontinence were also observed. A timeline of the symptom and progress was shown in Figure 3. Nocturnal SpO_2_ on ventilation support showed a transient drop to 70% during the first half of the night, before increasing to 95% by late midnight. An arterial blood gas test on room air revealed a pH of 7.36, PaCO_2_ of 55 mmHg, PaO_2_ of 72 mmHg, and HCO_3_^–^ of 31.2 mmol/L. On admission, acquired pulmonary hypertension was detected using echocardiography. Non-invasive positive pressure ventilation (NPPV) titration was performed at night with simultaneous PSG and TcPCO_2_, with IPAP/EPAP set to 19/6 cmH_2_O and frequency set to 20 times/min. PSG studies found that the patient could not trigger an IPAP/EPAP cycle during slow-wave sleep; therefore, the backup rate was adjusted to be relatively high (20 times per minute). We set the backup rate to 20 to ensure reliable trigger ventilation and adjusted the IPAP/EPAP to increase tidal volume. Airflow, tidal volume and leaks were monitored. No significant leakages were observed. The pressure support gradually increased with the tidal volume, SpO_2_, and TcPCO_2_ improved. The final titration pressure setting was IPAP/EPAP 21/8, supplemental oxygen (2 L/min), the SpO_2_ reached 95%, and TcPCO2 55–65 mmHg (similar to when she was awake). The patient tolerated the procedure well and did not experience nocturia. However, hypoxemia (SpO_2_ < 90%) and hypercapnia (TcPCO_2_ > 70 mmHg) still appeared during stage N3 of the first two sleep cycles (Supplementary Figure 1) without much change in ventilator settings. The progression of the disease was presumed to be related to further deterioration of the hypothalamus and dysfunction of the respiratory center.

## Discussion

In this case, we report the management of a 5-year-old girl with rapid and early onset obesity, alveolar hypoventilation, hypothalamic dysfunction (hyperprolactinemia, central hypothyroidism, GH deficiency), and behavioral disorders (irritability and aggression) in her first visit, presenting with convincing evidence of a ROHHAD diagnosis. This is the first reported case of ROHHAD in the Chinese population. ROHHAD syndrome is a life-threatening disease in children, with death occurring at an average age of approximately 10 years (3). Central hypoventilation caused by a deficit in breathing control is the most lethal feature of ROHHAD and carries a high risk of cardiorespiratory arrest, even during treatment. A systematic review of the clinical manifestations of ROHHAD by Lee et al. involved 46 studies with 158 patients; the calculated median age of onset was 4 years, and the prevalence of the disease in girls was twice that in boys (7). The diagnosis of ROHHAD was based on a cooperative consultation with multidisciplinary experts. Diagnosis is often missed or delayed, as in our case. Although the girl presented with rapid weight gain as early as 2 years of age, she was diagnosed at the age of 5. However, the effects of this delay on brain growth and development remain unknown.

Rapid-onset obesity with hypothalamic dysfunction, hypoventilation, and autonomic dysregulation syndrome may be clinically heterogeneous. Most commonly, the patient initially presents with dramatic and rapid weight gain associated with hyperphagia and varying degrees of hypoventilation and autonomic nervous system dysregulation. Hypothalamic dysfunction is manifested by high prolactin levels, low thyroid hormone levels, GH deficiency, and early or late puberty among other abnormalities. Dhondt et al. also reported a case of ROHHAD with a hypocretin-1 deficiency that displayed the classic features of narcolepsy with cataplexy, which broadened hypothalamus dysfunction and expands the phenotype of ROHHAD (8). A subset of patients (52.1%) with ROHHAD will develop neuroendocrine tumors, such as ganglioneuromas or ganglioneuroblastomas. Some researchers have recommended that this phenotype be termed ROHHADNET (7, 9).

Breathing abnormalities are present in almost all patients (2). Children may snore or show breathing pauses during sleep, without the perception of dyspnea, which can be easily ignored. All children with ROHHAD develop alveolar hypoventilation with very shallow breathing during sleep (both nap and night). In more severely affected patients with ROHHAD, hypoventilation is visible when they are not only asleep but also awake. In patients with ROHHAD, even if breathing is sufficient during the daytime, there is a lack of normal responsiveness to aggravated hypoxia and hypercapnia during sleep and wakefulness. Carroll et al. found that patients with ROHHAD showed a diminished tidal volume and inspiratory drive response to a hypoxic hypercapnia test, combined with a lack of behavioral perception of asphyxia, indicating a blunted chemosensory response (10). Our patient showed a progression to central hypoventilation. When challenged by a respiratory infection or anesthesia, these patients can suddenly develop respiratory failure and require mechanical ventilation (11, 12); as in our case, intubation and mechanical ventilation were required during an episode of pneumonia. According to published articles, the proportion of patients with ROHHAD receiving mechanical ventilation is 42–47%, and of those, NPPV is required in 53% of patients (2, 7). In a review of 51 patients with ROHHAD, 69% required artificial ventilation via tracheostomy, with a mean age of 3.8 years, and 31% required 24-h NPPV support (13). Recently, Reppucci et al. reviewed six children with suspected ROHHAD with a median age of 7.2 years of nocturnal hypoventilation onset; five of six patients accepted BPAP without tracheostomy, which is similar to our study results (14). Historically, invasive ventilation through tracheostomy has been the first line of treatment. However, with the development of sleep medicine, non-invasive ventilation with both frequency and volume backup mode has become more available, and treatment during sleep can improve wake-time ventilation or delay the progression of the disease. Undiagnosed or untreated children with ROHHAD may present with complications of chronic hypoventilation, including pulmonary hypertension, cor pulmonale, seizures, or developmental delays. Once a diagnosis is suspected, a full respiratory assessment is required during wakefulness and sleep, and BPAP titration may be performed. Although the patient in this case report underwent several PSG tests and BPAP titrations, chronic hypoventilation was difficult to completely eliminate because of the inconsistent use of BPAP and blunted chemosensory response.

In the present case report, hypoventilation due to ROHHAD was severe during NREM sleep, especially in stage N3 of the first two sleep cycles, showing frequent and significant oxygen desaturation and CO_2_ retention. This phenomenon is in contrast to other sleep-related breathing disorders, such as obstructive sleep apnea, and other sleep-related hypoventilations, which are often related to REM (15). Some studies have reported that hypoventilation in CHS/LO-CHS is more apparent in NREM sleep than in REM sleep, with a smaller increase in respiratory rate, greater decrease in minute ventilation, infrequent arousal, and frequent central sleep apnea during NREM sleep than during REM sleep (16, 17). Goldbart et al. found that children with ROHHAD syndrome have suppressed slow-wave activity power and shallower slow-wave activity slopes during the first two sleep cycles, which may explain the hypoventilation in stage N3 during the first half of the night (18). In addition, previous reports have indicated that hypothyroidism contributes to central hypoventilation, and obvious hypoxic and hypercarbia disorders are observed in untreated thyroid dysfunction (19). Therefore, for patients with ROHHAD, continuous hypothyroidism treatment and regular evaluation of thyroid function are required. Annual echocardiography, ambulatory cardiac monitoring, neurocognitive evaluations, urine tests, and imaging of neural crest tumors are also needed. To ensure optimal oxygenation and ventilation, continued patient education is needed to improve PAP adherence and PSG must be repeated every 3–6 months during follow-up. Anesthesia is challenging in patients with ROHHAD. For anesthetic management of patients with ROHHAD, Chandrakantan and Poulton reported the use of short-acting anesthetics with minimal respiratory effects combined with anxiolytics. Continuous electrocardiography, SpO_2_, and end-tidal carbon dioxide monitoring should be performed even with conscious sedation in brief procedures (20).

The etiopathogenesis of ROHHAD is unknown; however, an immune-mediated process is suspected in some cases. Several reports of immunoglobin therapy or immunosuppressive drugs (rituximab and cyclophosphamide) in patients with ROHHAD report a transient or partial improvement, but the target and long-term efficacy are unclear (21–23). These therapies were not prescribed to our patients as there was insufficient evidence of their value.

In conclusion, we described the presentation and chronic management of a patient with ROHHAD with non-invasive mechanical ventilation at the time of diagnosis. We believe that early recognition and application of NPPV during sleep is vital to slow the deterioration of respiratory function and improve the quality of life. Multidisciplinary care is crucial for the successful diagnosis and management of these patients.

## Data availability statement

The original contributions presented in this study are included in the article/Supplementary material, further inquiries can be directed to the corresponding authors.

## Ethics statement

The studies involving human participants were reviewed and approved by the Ethics Review Committee of Peking University. Written informed consent to participate in this case study was provided by the participants’ legal guardian.

## Author contributions

XD, ZG, and FH contributed to the study concept and design and reviewed the manuscript. RZ collected the medical information of the participant and completed draft manuscript. XD was the guarantor of the manuscript and took responsibility for the integrity of the data. All authors contributed to the article and approved the submitted version.
