# Supplementary material for: Case Report: Considerations of nocturnal ventilator support in ROHHAD syndrome in chronic care of childhood central hypoventilation with hypothalamus dysfunction
Source: Front Pediatr. 2022 Aug 31;10:919921. doi: 10.3389/fped.2022.919921 (PMC9470944; doi:10.3389/fped.2022.919921)
Supplement: Supplementary file 1 [file Data_Sheet_1.PDF]

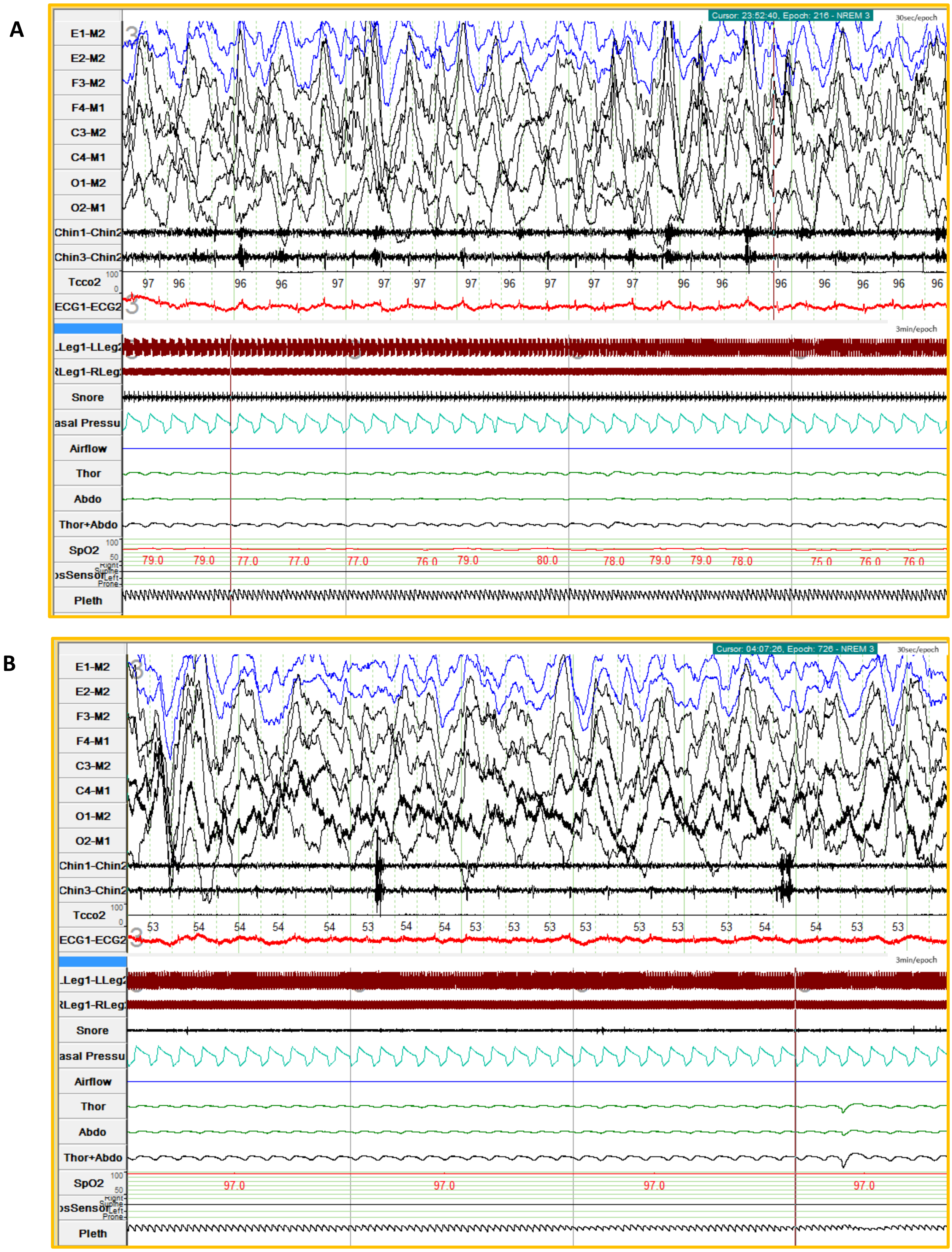

Supplementary FigureS1 PSG under BPAP titration of the girl at 10 years of age during different N3 sleep cycle.

A: The first cycle of N3 sleep: With BPAP ventilation (IPAP/EPAP 19/6cmH$_2$O, f 20 times/min), there was no sleep apnea and hypopnea events, hypoxemia (SpO$_2$ 75-80%) and hypercapnia (TcPCO$_2$ 96 mmHg) still appeared in the first cycle of N3 sleep stage.

B: The forth cycle of N3 sleep: Neither hypoxemia nor hypercapnia was found in the fourth cycle of N3 sleep at the same BPAP level.

Electroencephalogram (F3M2, F4M1, C3M2, C4M1, O1M2, and O2M1), electrooculogram (E1M2, E2M2), , chin muscle electromyogram(Chin1-Chin2, Chin3-Chin2); L leg and R leg: electromyogram of left and right leg, respectively; ECG: electrocardiogram, TcCO2: transcutaneous carbon dioxide analysis; Nasal Pressure: pressure flow of oral-nasal mask; SpO_2 = oxygen saturation, Thor - chest, Abdo - abdomen, PosSensor - body position, Plethysmog = plethysmography.
